# Supplementary material for: Fungal parasites infecting N2-fixing cyanobacteria reshape carbon and N2 fixation and trophic transfer
Source: Nat Commun. 2026 Jan 2;17:154. doi: 10.1038/s41467-025-67818-x (PMC12775406; doi:10.1038/s41467-025-67818-x)
Supplement: Supplementary file 1 — Supplementary Information [file 41467_2025_67818_MOESM1_ESM.pdf]

Supplementary Information

Fungal parasites infecting N_2-fixing cyanobacteria reshape carbon and N_2 fixation and trophic transfer

Anna Feuring, Connor D. Lawrence, Jessica Salcedo, Martin J. Whitehouse, Angela Vogts, Luca Zoccarato, Isabell Klawonn

Supplementary Table S1. Biogeochemical parameters during isotope-tracer incubations. N.d. – not detectable (below detection limit). Data are listed as mean±sd (N – number of replicate bottles).

Parameter	Time point [h]	Value	N
PO_4^{3-} concentration µmol L^{-1}	0	0.23±0.06	4
	10	0.24±0.03	4
	21	0.14±0.02	4
NO_3^- concentration µmol L^{-1}	0	n.d. (<0.20)	4
	10	0.35±0.12	4
	21	n.d. (<0.20)	4
NO_2^- concentration µmol L^{-1}	0	n.d. (<0.05)	4
	10	0.051±0.001	4
	21	n.d. (<0.05)	4
NH_4^+ concentration µmol L^{-1}	0	0.65±0.15	4
	10	0.76±0.13	4
	21	n.d. (<0.50)	4
Dissolved Organic Carbon (DOC) µmol L^{-1}	0	356±22	4
	10	376±11	4
	21	360±6	4
Dissolved Nitrogen (DN) µmol L^{-1}	0	15.6±0.6	4
	10	16.2±0.6	4
	21	15.6±0.6	4
Particulate Organic Carbon (POC) µmol L^{-1}	0	25.6±1.5	4
	10	23.7±2.0	4
	21	28.6±0.6	4
Particulate Organic Nitrogen (PON) µmol L^{-1}	0	3.5±0.2	4
	10	3.3±0.2	4
	21	3.8±0.1	4
POC:PON ratio mol:mol	0	7.4±0.5	4
	10	7.2±0.2	4
	21	7.6±0.2	4
Carbon fixation rate µmol C d^{-1} L^{-1}	0–21 (10 h dark + 11 h light)	6.1±0.1	3
N_2 fixation rate µmol N d^{-1} L^{-1}	0–21 (10 h dark + 11 h light)	0.22±0.01	3

Supplementary Table S2. Phytoplankton community composition. Cell abundances were used to calculate the carbon biomass of the dominating autotrophic and mixotrophic phytoplankton according to Svedén, et al. [1], HELCOM [2], and Olenina, et al. [3]. Data are listed as mean±sd (N=12 replicate incubation bottles).

	Cell abundances cells mL^{-1}	Carbon biomass µmol C L^{-1}	Carbon biomass % of total phytoplankton
**Cyanobacteria**			
Dolichospermum	2298±497	2.0±0.4	34%
Anabaena	16±4	0.009±0.002	0.1%
Chroococcales	13073±3217	0.31±0.06	5%
Sum Cyanobacteria	15387±3660	2.3±0.5	40%
**Diatoms**			
Dactyliosolen fragilissimus	76±10	2.3±0.3	39%
Cyclotella	52±10	0.20±0.04	3%
Skeletonema marinoi	2±2	0.002±0.002	0.03%
Chaetoceros	2±1	0.04±0.03	1%
Thalassionema nitzschioides	3±2	0.01±0.00	0.2%
Sum Diatoms	137±9	2.5±0.3	44%
**Dinoflagellates**			
Dinophyceae	25±6	0.65±0.19	11%
Tripos muelleri	0.2±0.1	0.23±0.12	4%
Sum Dinoflagellates	26±6	0.87±0.23	15%
**Others**			
Cymbomonas tetramitiformis	4±1	0.04±0.01	1%
Binuclearia lauterbornii	45±19	0.02±0.01	0.3%
Dinobryon	33±10	0.02±0.01	0.3%
Sum Others	81±25	0.08±0.02	1%

Supplementary Table S3. Abundance and infection prevalence of *Dolichospermum* filaments and cells during isotope-tracer incubations. Data are listed as mean±sd (N=12 replicate incubation bottles).

**Counting unit**	**Abundance [mL^{-1}]**	**Prevalence [%]**
Filaments	88±13	57±6
Vegetative cells	2097±244	5±3
Heterocytes	165±24	44±8
Akinetes	36±14	82±12
Total cells	2298±270	9±3

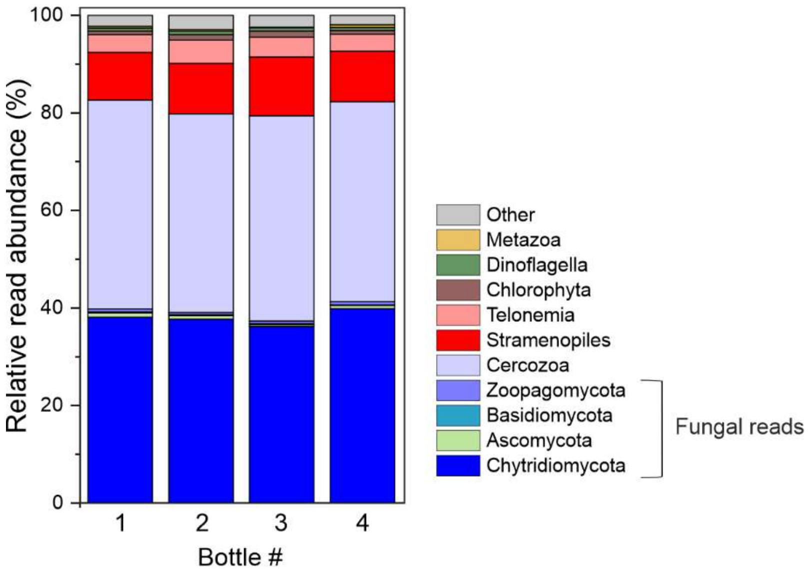

Supplementary Figure S1 Eukaryote community composition. The community composition is shown as the relative abundance of ASV counts (18S rRNA gene-based, PR2 reference database) for the four replicate bottles. The co-amplification of Stramenopiles was blocked using an annealing-blocking oligonucleotide.

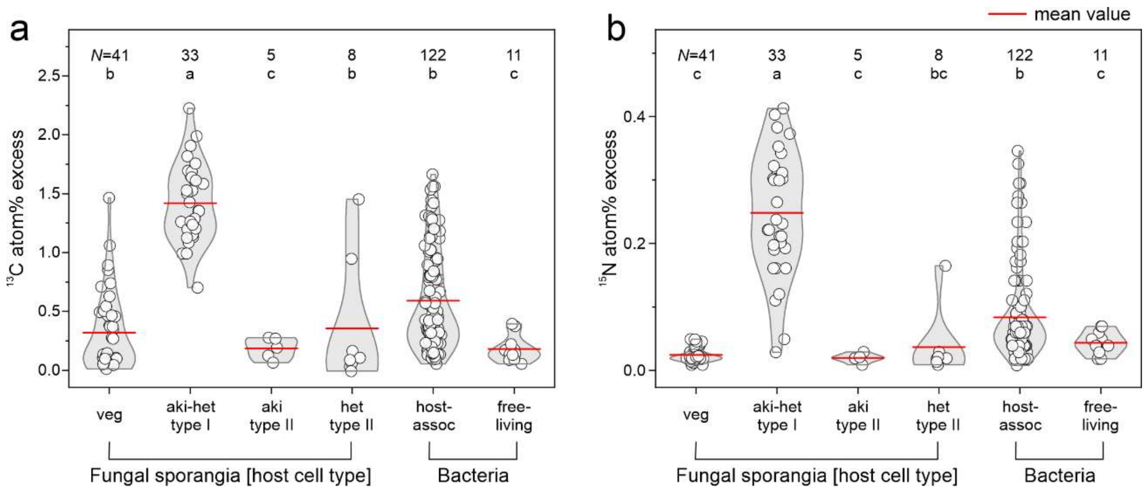

Supplementary Figure S2. ^{13}C and ^{15}N atom% excess measured in fungal sporangia and bacteria. Fungal sporangia represent mature sporangia. Single data points are shown as white circles. Distribution curve type: kernel smooth. The letters a–c denote significantly different groups (^{13}C at% excess: Kruskal–Wallis test, two-sided: $H(5) = 93.6$, $p = 1.16\times10^{-18}$, effect size $\eta^2(H) = 0.42$ (95% CI: 0.33–0.50), $N = 220$; ^{15}N at% excess: Kruskal–Wallis test, two-sided: $H(5) = 118.0$, $p = 8.51\times10^{-24}$,

effect size $\eta^2(H) = 0.53$ (95% CI: 0.43–0.63), $N = 220$; N is the number of analyzed cells). Source data are provided as a Source Data file.

Supplementary Table S4. ^{13}C and ^{15}N incorporation and growth rates for cyanobacterial host cells, bacteria, and fungal sporangia.

Analyzed cell type	Infection	Host cell type	Sporangium development stage	^{13}C atom% excess	C-specific C-growth d^{-1}	C-incorporation rate pmol C $cell^{-1}$ d^{-1}	^{15}N atom% excess	N-specific N-growth d^{-1}	N-incorporation rate pmol N $cell^{-1}$ d^{-1}	C/N incorporation ratio mol:mol	*N*
Host	ui	Vegetative	-	1.42±0.26	0.52±0.11	0.39±0.08	0.176±0.069	0.36±0.16	0.042±0.019	11.6±9.0	3527
Host	ui	Heterocyte type I	-	0.41±0.19	0.13±0.06	0.10±0.05	0.078±0.042	0.14±0.09	0.018±0.011	6.1±2.3	55
Host	ui	Heterocyte type II	-	0.57±0.23	0.18±0.08	0.14±0.06	0.115±0.056	0.22±0.12	0.028±0.015	5.7±2.1	85
Host	ui	Akinete type I	-	1.55±0.30	0.58±0.14	4.50±1.11	0.249±0.113	0.58±0.37	0.712±0.450	8.3±4.4	104
Host	i	Vegetative	Encystment	1.44±0.19	0.52±0.09	0.39±0.06	0.183±0.052	0.37±0.12	0.043±0.014	10.1±4.4	67
Host	i	Heterocyte type I	Encystment	0.45±0.21	0.14±0.07	0.11±0.06	0.072±0.019	0.13±0.04	0.016±0.005	6.9±3.2	7
Host	i	Heterocyte type II	Encystment	0.70±0.40	0.23±0.15	0.18±0.11	0.129±0.059	0.25±0.13	0.031±0.016	6.1±2.6	31
Host	i	Akinete type I	Encystment	1.60±0.27	0.60±0.13	4.66±1.03	0.286±0.126	0.71±0.47	0.868±0.578	6.7±2.7	10
Host	i	Vegetative	Matured	1.41±0.31	0.52±0.14	0.38±0.10	0.183±0.067	0.38±0.17	0.044±0.020	9.6±3.3	59
Host	i	Heterocyte type I	Matured	0.44±0.20	0.14±0.07	0.11±0.05	0.081±0.037	0.15±0.07	0.019±0.009	6.0±1.8	26
Host	i	Akinete type I	Matured	1.29±0.29	0.46±0.12	3.57±0.91	0.131±0.072	0.26±0.16	0.318±0.202	14.7±7.2	26
Host	i	Vegetative	Post-infection	1.32±0.25	0.47±0.11	0.35±0.08	0.166±0.079	0.34±0.18	0.040±0.022	11.6±7.0	8
Host	i	Heterocyte type I	Post-infection	0.32±0.16	0.10±0.05	0.08±0.04	0.055±0.037	0.10±0.08	0.013±0.010	7.1±3.5	62
Host	i	Akinete type I	Post-infection	1.18±0.38	0.42±0.15	3.22±1.19	0.129±0.072	0.26±0.17	0.313±0.208	12.9±7.0	65
Sporangium	-	Vegetative	Mature	0.32±0.33	-	-	0.024±0.011	-	-	-	41
Sporangium	-	Heterocyte-akinete type I	Mature	1.42±0.33	-	-	0.248±0.096	-	-	-	33
Sporangium	-	Heterocyte type II	Mature	0.35±0.54	-	-	0.037±0.052	-	-	-	8
Sporangium		Akinete type II	Mature	0.19±0.09	-	-	0.019±0.007	-	-	-	5
Bacteria assoc.	-	-	-	0.59±0.42	-	-	0.083±0.070	-	-	-	122
Bacteria free-living	-	-	-	0.18±0.11	-	-	0.043±0.018	-	-	-	11
Control cells	-	-	-	-0.02±0.02	-	-	0.010±0.007	-	-	-	475

Fungal sporangia were differentiated based on the host cell type, infection status (ui – un-infected, i – infected), and sporangial development stage. The sporangial development stages included the (1) onset of infections represented by encysted zoospores, (2) ongoing infection represented by matured sporangia, and (3) post-infection represented by empty, collapsed sporangia. Associated bacteria represent those located in close proximity (<10 µm) to the cyanobacterial cell. Control cells were incubated without isotopically-labelled substrates. Growth rates and incorporation rates were calculated exclusively for cyanobacterial host cells since the isotope label% in the substrate pool is required for the calculations, and it is known only for the primary consumers (^{13}C- and $^{15}N_2$-fixing cells) but not for secondary consumers (feeding on carbon and nitrogen originating from carbon and N_2-fixing cells). N – number of analysed cells. The low N (≤10) for some cell types is explained by their rare occurrence on our filter samples (associated mean values should thus be considered with care due to the small sample sizes).

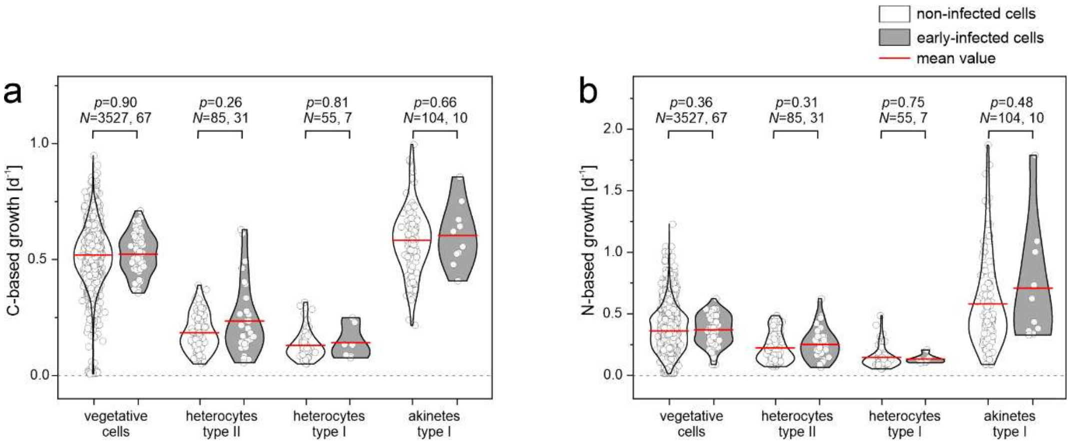

Supplementary Figure S3. Carbon and nitrogen-based growth rates of non-infected and early infected *Dolichospermum* cells. Early infected cells were identified by their newly encysted zoospore. The p-values resulted from two-sided two-sample comparisons (two-sample Student's t-test for normal distribution with equal variance, and Wilcoxon rank-sum/Mann–Whitney for non-normal distribution). Statistics: vegetative cells, C-based growth: W = 119251, p = 0.896, rank-biserial r = 0.009, 95% CI = –0.13 to 0.15; N-based growth: W = 125796.5, p = 0.364, rank-biserial r = 0.06, 95% CI = –0.07 to 0.20; heterocytes type II, C-based growth: W = 1499, p = 0.259, rank-biserial r = 0.14, 95% CI = –0.10 to 0.36; N-based growth: W = 1479, p = 0.314, rank-biserial r = 0.12, 95% CI = –0.11 to 0.35; heterocytes type I, C-based growth: W = 204, p = 0.807, rank-biserial r = 0.06, 95% CI = –0.38 to 0.48; N-based growth: W = 207, p = 0.754, rank-biserial r = 0.08, 95% CI = –0.36 to 0.49; and akinetes type I, C-based growth: t(112) = 0.44, p = 0.658, Cohen's d = 0.15, 95% CI = –0.50 to 0.80; N-based growth: W = 591, p = 0.480, rank-biserial r = 0.14, 95% CI = –0.23 to 0.47. The numbers of analyzed cells are given as *N*. Single data points are shown as white circles. Distribution curve type: kernel smooth. Source data are provided as a Source Data file.

Supplementary Note S1. Fungal infections on *Dolichospermum*, *Nodularia*, and *Aphanizomenon* in the Baltic Sea. In addition to our sampling site in the Southern Baltic Sea (Heiligendamm, HD), we collected samples on transects from the coastal to the open Baltic Sea (Baltic Sea Monitoring Program) during summer in 2022–2024. Depth-integrated samples were collected from the upper water column with a vertical plankton net (10 µm mesh size, Apstein 50, Hydro-Bios GmbH, Germany) from 0–10 m, preserved with Lugol's solution (pH=7) and stored in 100 mL brown glass bottles at

4°C. Using microscopy, we searched for the three filamentous N_2-fixing cyanobacteria *Dolichsopermum*, *Nodularia,* and *Aphanizomenon*. Their presence and, if present, their infection prevalence in filaments were recorded.

Fungal infections in *Dolichospermum* were most prominent during the summers of 2022 and 2023, with maximum prevalences reaching up to 56% of filaments, whereas infections were mostly absent in the summer of 2024. In *Nodularia*, putative infections—characterized by fungi-like structures—were observed in 7 out of 10 sampling weeks during summer 2024, with infection rates reaching up to 71% of filaments, and sporangia being mostly associated with the heterocytes. In contrast, such infections were rarely detected in 2022 and 2023. The parasite morphology in *Nodularia* was distinctly different from that observed in *Dolichospermum*. Sporangia appeared smaller and hyphae-like (tubular) rhizoids extended through the *Nodularia* filaments, whereas only fine rhizoids extended inside *Dolichospermum* filaments (Fig. 6). *Aphanizomenon* was the least affected across all three years. On the rare occasions when infections were detected, sporangia were exclusively found on heterocytes (Fig. 6a, b). Similar infections have recently also been observed in the Northern Baltic Sea [4].

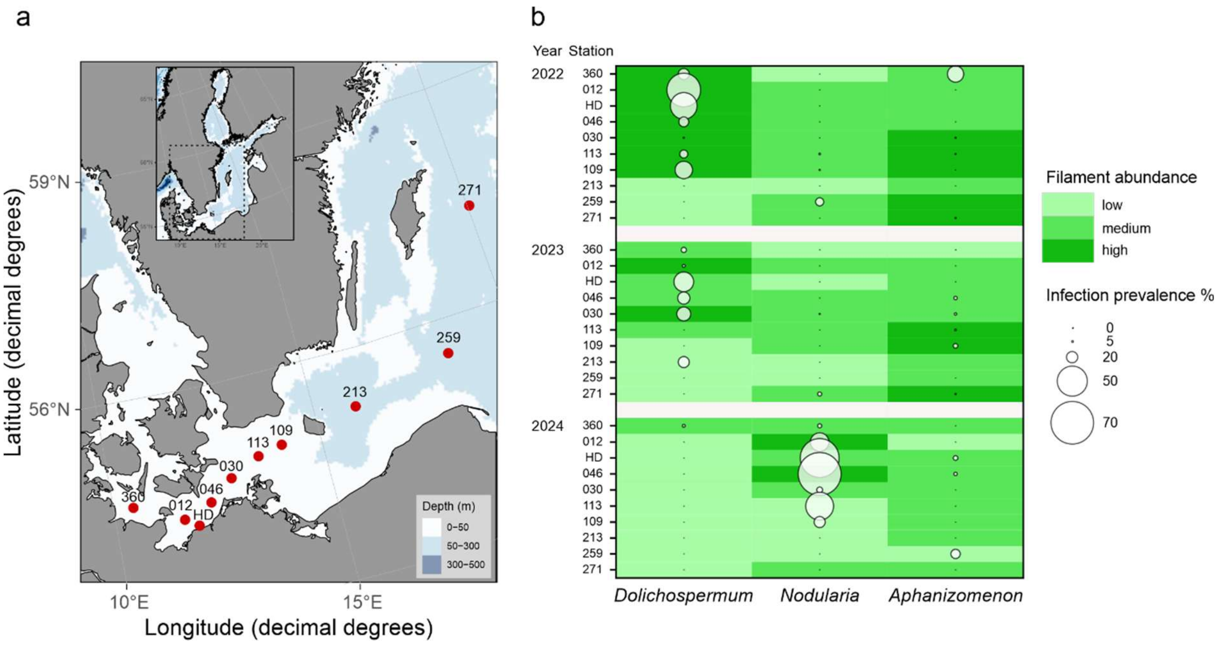

Supplementary Figure S4. Fungal infection prevalence on *Dolichospermum*, *Nodularia*, and *Aphanizomenon* in the Baltic Sea. (a) Samples were collected along a transect from the South-Western Baltic Sea (station 360) to the central Baltic Sea (station 271) during summer 2022–2024 (week 31 and/or 32). The entire Baltic Sea

map is shown on the insert, with the sampling region framed with a dashed line. The maps were created in RStudio using the packages ggOceanMaps [5], ggplot2 [6], and sf [7,8]. HD – Heiligendamm sampling station. **(b)** Abundance of cyanobacterial filaments (color-coded) and infection prevalence (% of filaments, bubble-size). Filaments were counted from plankton net samples. Such samples do not allow to report abundance data (e.g. as filaments mL^{-1}), and thus we broadly categorized abundances as *low*, *medium*, and *high*. Source data are provided as a Source Data file.

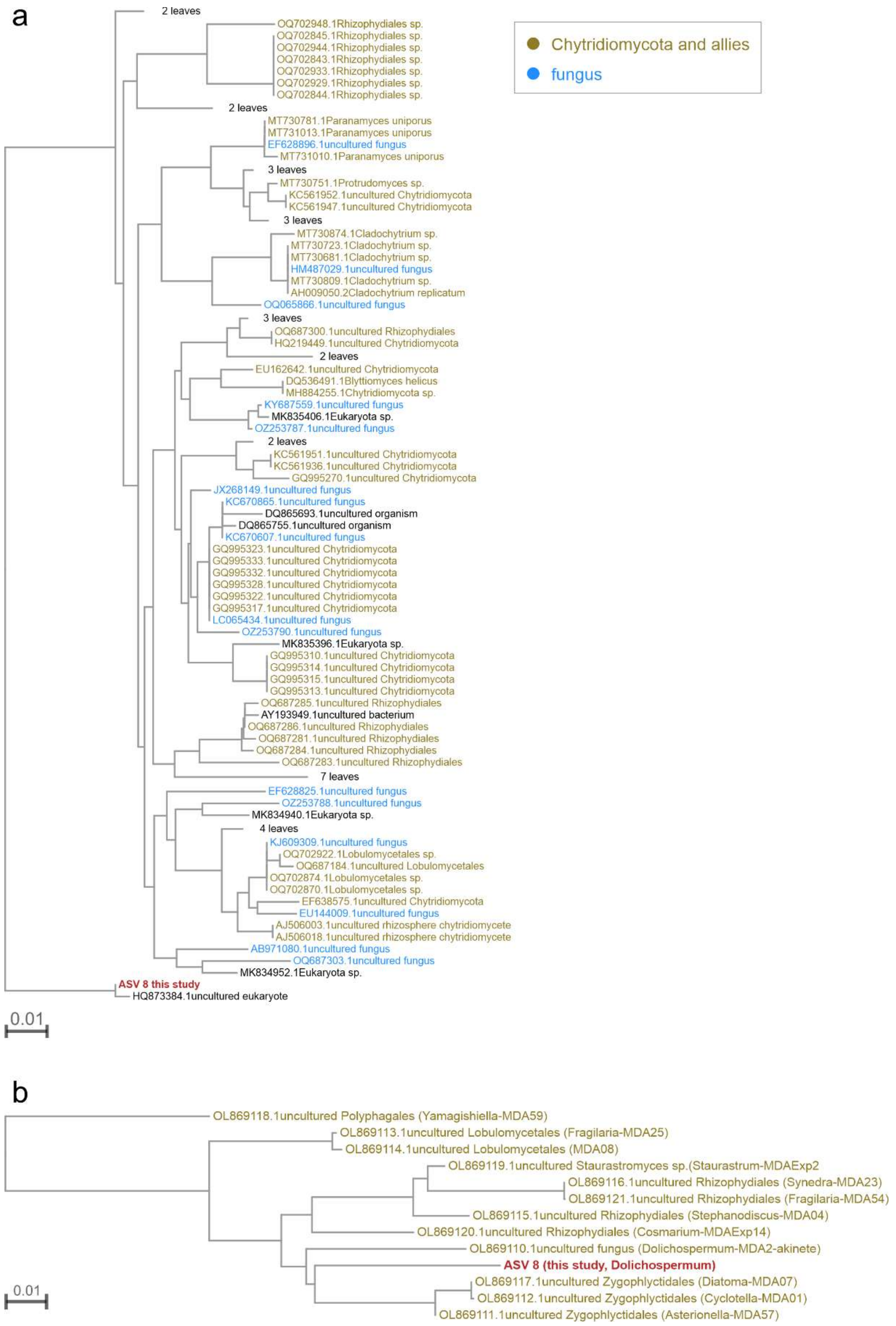

Supplementary Figure S5. Phylogeny of fungal parasite. Phylogenetic trees (non-curated) created on NCBI using **(a)** the 100 best BLAST matches and **(b)** sequences of chytrids known to infect phytoplankton data obtained from [9]. ASV 8 (shown in red) represents the most abundant fungal ASV in our sample (87% of Chytridiomycota-assigned reads).

Supplementary Table S5. Accession numbers and reported taxon used to built the phylogenetic tree in Supplementary Figure S5. Accessions were retrieved from NCBI (National Center for Biotechnology Information).

Tree (a)	
Accession	Taxon
OQ702948.1	Rhizophydiales sp.
OQ702845.1	Rhizophydiales sp.
OQ702944.1	Rhizophydiales sp.
OQ702843.1	Rhizophydiales sp.
OQ702933.1	Rhizophydiales sp.
OQ702929.1	Rhizophydiales sp.
OQ702844.1	Rhizophydiales sp.
MT730781.1	*Paramanyces uniporus*
MT731013.1	*Paramanyces uniporus*
EF628896.1	uncultured fungus
MT731010.1	*Paramanyces uniporus*
MT730751.1	*Protrudomyces* sp.
KC561952.1	uncultured Chytridiomycota
KC561947.1	uncultured Chytridiomycota
MT730874.1	*Cladochytrium* sp.
MT730723.1	*Cladochytrium* sp.
MT730681.1	*Cladochytrium* sp.
HM487029.1	uncultured fungus
MT730809.1	*Cladochytrium* sp.
AH009050.2	*Cladochytrium replicatum*
OQ065866.1	uncultured fungus
OQ687300.1	uncultured Rhizophydiales
HQ219449.1	uncultured Chytridiomycota
EU162642.1	uncultured Chytridiomycota
DQ536491.1	*Blyttioymyces helicus*
MH884255.1	Chytridiomycota sp.
KY678559.1	uncultured fungus
MK835406.1	Eukaryota sp.
OZ253787.1	uncultured fungus
KC561951.1	uncultured Chytridiomycota
KC561936.1	uncultured Chytridiomycota
GQ995270.1	uncultured Chytridiomycota
JX268149.1	uncultured fungus
KC670865.1	uncultured fungus
DQ865693.1	uncultured organism
DQ865755.1	uncultured organism
KC670607.1	uncultured fungus
GQ995323.1	uncultured Chytridiomycota
GQ995333.1	uncultured Chytridiomycota
GQ995332.1	uncultured Chytridiomycota
GQ995328.1	uncultured Chytridiomycota
GQ995322.1	uncultured Chytridiomycota
GQ995317.1	uncultured Chytridiomycota
LC065434.1	uncultured fungus
OZ253790.1	uncultured fungus
MK835396.1	Eukaryota sp.
GQ995310.1	uncultured Chytridiomycota
GQ995314.1	uncultured Chytridiomycota
GQ995315.1	uncultured Chytridiomycota
GQ995313.1	uncultured Chytridiomycota
OQ687285.1	uncultured Rhizophydiales
AY193949.1	uncultured bacterium
OQ687286.1	uncultured Rhizophydiales
OQ687281.1	uncultured Rhizophydiales
OQ687284.1	uncultured Rhizophydiales
OQ687283.1	uncultured Rhizophydiales
EF628825.1	uncultured fungus

OZ253788.1	uncultured fungus
MK834940.1	Eukaryota sp.
KJ609309.1	uncultured fungus
OQ702922.1	Lobulomycetales sp.
OQ687184.1	uncultured Lobulomycetales
OQ702874.1	Lobulomycetales sp.
OQ702870.1	Lobulomycetales sp.
EF638575.1	uncultured Chytridiomycota
EU144009.1	uncultured fungus
AJ506003.1	uncultured rhizosphere chytridiomycete
AJ506018.1	uncultured rhizosphere chytridiomycete
AB971080.1	uncultured fungus
OQ687303.1	uncultured fungus
ASV8	this study
HQ873384.1	uncultured eukaryote
MK834952.1	Eukaryota sp.
Tree (b)	
accession	taxon (host in brackets)
OL869118.1	uncultured Polyphagales (*Yamagishiella*-MDA59)
OL869113.1	uncultured Lobulomycetales (*Fragilaria*-MDA25)
OL869114.1	uncultured Lobulomycetales (MDA08)
OL869119.1	uncultured Staurastromyces sp. (*Staurastrum*-MDAExp2)
OL869116.1	uncultured Rhizophydiales (*Synedra*-MDA23)
OL869121.1	uncultured Rhizophydiales (*Fragilaria*-MDA54)
OL869115.1	uncultured Rhizophydiales (*Stephanodiscus*-MDA04)
OL869120.1	uncultured Rhizophydiales (*Cosmarium*-MDAExp14)
OL869110.1	uncultured fungus (*Dolichospermum*-MDA2-akinete)
ASV8	this study (*Dolichospermum*)
OL869117.1	uncultured Zygophycitidales (Diatoma-MDA07)
OL869112.1	uncultured Zygophycitidales (*Cyclotella*-MDA01)
OL869111.1	uncultured Zygophycitidales (*Asterionella*-MDA57)

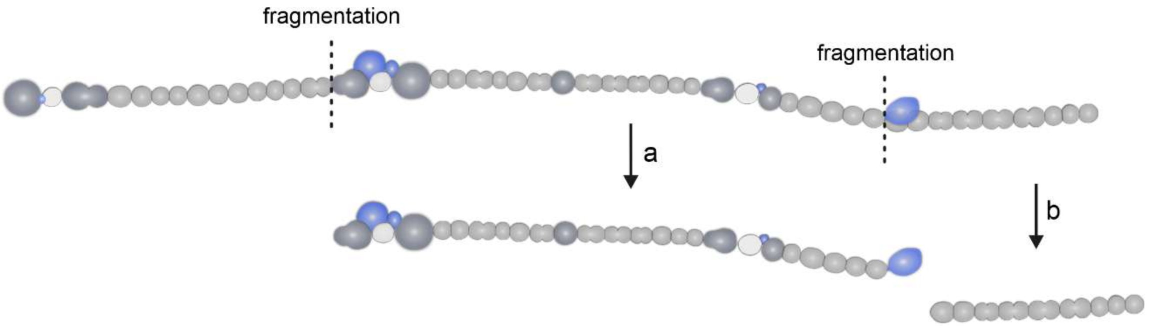

Supplementary Figure S6. Hypothetical fragmentation process in *Dolichospermum* filaments. Cells with mature sporangia were identified as likely sites of filament breakage. Fragmentation at these points generates two types of filament fragments: infected fragments bearing apical sporangia **(a)**, and shorter non-infected fragments **(b)**. This mechanism may explain why the majority (62%) of infected filaments observed contained apical sporangia.

Supplementary Table S6. Cell dimensions, volumes, as well as carbon and nitrogen contents of the various *Dolichospermum* cell types, fungal sporangia, and bacteria. Cell dimensions were measured from PFA-preserved cells on filters, and used to calculate the cell volume assuming an ellipsoid shape (V=4π/3 × Length/2 × Height/2 × Height/2). The carbon content was calculated assuming 0.21 pg C μm^{-3} (or 0.017 pmol C μm^{-3}) following Svedén, et al. [1] who determined this biomass conversion factor for Baltic Sea cyanobacteria. A C:N ratio of 6.3 was applied as also reported by Svedén, et al. [1] and close to the herein measured bulk POC:PON ratio of 7.4±0.4 (N=12 incubation bottles). For simplicity, the same biomass conversion factor was also applied to fungal sporangia. For akinetes, a 1.25-fold higher C and N content was applied since akinetes are known to store C-rich glycogen and N-rich phycobiliproteins [10]. The bacterial biomass was calculated assuming 0.032 pmol C μm^{-3} and a C:N ratio (mol:mol) of 4.3 following Lee and Fuhrman [11].

Cell type	Length [µm]	Height [µm]	Cell volume [μm^3]	C content (pmol C $cell^{-1}$)	N content (pmol N $cell^{-1}$)	*N*
Vegetative	4.8 ± 0.8	4.0 ± 0.5	42 ± 17	0.7 ± 0.3	0.12 ± 0.05	50
Heterocytes	5.3 ± 0.7	3.9 ± 0.6	45 ± 18	0.8 ± 0.3	0.13 ± 0.05	50
Akinetes	10.4 ± 2.9	7.5 ± 1.7	354 ± 271	7.7 ± 5.9	1.23 ± 0.94	50
Mature sporangia	8.7 ± 1.3	7.8 ± 1.1	294 ± 119	5.1 ± 2.1	0.82 ± 0.33	50
Early sporangium (encysted zoospores)	1.7 ± 0.3	1.6 ± 0.2	2.3 ± 1.0	0.04 ± 0.02	0.006 ± 0.003	50
Heterotrophic bacteria	0.45*	0.60*	0.06	0.0020	0.0005	*

* Bacterial cell dimensions were derived from Klawonn, et al. [12] who measured bacterial sizes during cyanobacterial blooms in summer at a coastal station in the Baltic Sea, similar to our sampling location.

Supplementary Note S2. Extrapolating carbon and nitrogen incorporation and nitrogen transfer to total community level

Using single-cell carbon and nitrogen incorporation rates (Supplementary Table S4) combined with cell count and biomass data, we extrapolated cyanobacterial carbon and N_2 fixation and the fate of newly fixed nitrogen within the microbial food web during fungal epidemics. The set parameters are listed in Supplementary Table S7.

The abundance of *Dolichospermum* cells was set at 2.3×10^6 cells L^{-1}, based on counts from the incubation water. During the incubations, infection prevalence reached 9% of the total *Dolichospermum* population, with host specificity distributed as 1:9.6:17.9 across vegetative cells (4.6% infected), heterocytes (44.1%), and akinetes (82.2%). To maintain this host-specificity pattern, the same ratio was applied across the full range

of extrapolated prevalences from 0 to 11% of total cells. A maximum infection prevalence of 11% was set, as this corresponds to complete (100%) infection of akinetes (Supplementary Table S8). In natural conditions, however, fungal parasites may shift to less preferred host cells—such as vegetative cells and heterocytes—potentially resulting in overall infection prevalences exceeding 11%.

The nitrogen transfer to *Others* was defined as the difference between total N_2 fixation (as measured on bulk PON filters using EA-IRMS, see Supplementary Table S1) and the summed fraction assigned to bacteria, sporangia, and *Dolichospermum* cells. The extrapolation refers to the fixation and transfer per day.

Supplementary Table S7. Cell-specific biomass, growth rates and incorporation rates.

Cell-specific biomass

	C biomass	N biomass	Reference
	pmol C cell^{-1}	pmol N cell^{-1}	
Fungal sporangia - mature	5.14	0.82	Table S5
Fungal sporangia - encysted	0.040	0.006	Table S5
Bacteria - cell-associated	0.0020	0.0005	Table S5
Bacteria free-living	0.0020	0.0005	Table S5
Vegetative cells	0.74	0.12	Table S5
Heterocytes	0.79	0.13	Table S5
Akinetes	7.73	1.23	Table S5

Cell-specific growth rates

	C-based growth rate		N-based growth rate		Reference
	d^{-1}		d^{-1}		
Fungal sporangia (vegetative cell)	0.26		0.11		* see below
Fungal sporangia (akinete–heterocyte type I)	1.15		1.15		* see below
Fungal sporangia (heterocyte type II)	0.29		0.17		* see below
Bacteria - cell-associated	0.27		0.27		Klawonn, et al. [12]
Bacteria - free-living	0.09		0.09		Klawonn, et al. [12]
	non-infected	infected	non-infected	infected	
Vegetative cells	0.52	0.49	0.36	0.36	Table S4, † see below
Heterocytes	0.16	0.12	0.18	0.12	Table S4, † see below
Akinetes	0.58	0.44	0.58	0.26	Table S4, † see below

Cell-specific incorporation rates

	C incorporation	N incorporation	Reference
	pmol C cell^{-1} d^{-1}	pmol N cell^{-1} d^{-1}	
Fungal sporangia – vegetative cell	1.33	0.09	‡ see below
Fungal sporangia – akinete–heterocyte type I	5.93	0.94	‡ see below
Fungal sporangia – heterocyte type II	1.48	0.14	‡ see below
Bacteria - cell-associated	0.00055	0.00013	‡ see below
Bacteria free-living	0.00018	0.00004	‡ see below

	non-infected	infected	non-infected	infected	
Vegetative cells	0.39	0.37	0.04	0.04	Table S4
Heterocytes	0.12	0.09	0.02	0.02	Table S4
Akinetes	4.50	3.40	0.71	0.32	Table S4

* We isolated the *Dolichospermum*-fungus co-system from the sampling station (Heiligendamm) and monitored the infecting cycle. It lasted ca. 3 days from zoospore encystment to zoospore release, while approx. 10 zoospores (as a conservative estimate) were released per matured sporangium. This 3-day life cycle completion agrees with previous observations in natural populations [13]. The growth rate (proportional biomass increase per day) was thus set to 1.15 d^{-1} ($e^{\ln(10)/3}-1=1.15$). This maximum growth rate was assumed for zoospores infecting type I akinete–heterocyte junctions. Growth rates for zoospores encysting on vegetative cells and type II heterocytes were assumed to be 4-times lower for C-based growth rates for both cell types, and 10- and 7-times lower for N-based growth rates, respectively, as indicated by the difference in ^{13}C and ^{15}N APE in mature sporangia infecting the respective host cell types (Supplementary Table S4).

† Growth rates of infected host cells represent the mean of cells with mature and empty sporangia.

‡ Incorporation rates were calculated as Biomass × Growth rates (e.g., for carbon: C-biomass × C-based growth rate)

Supplementary Table S8. Prevalence, total and for each host cell type, used for extrapolating the N transfer to total community level

Prevalence				Reference
% of total *Dolichospermum* cells	% of vegetative cells	% of heterocytes	% of akinetes	
0%	0%	0%	0%	ratio 1 : 9.6 : 17.9
1%	1%	5%	9%	ratio 1 : 9.6 : 17.9
2%	1%	10%	18%	ratio 1 : 9.6 : 17.9
3%	2%	15%	27%	ratio 1 : 9.6 : 17.9
4%	2%	20%	37%	ratio 1 : 9.6 : 17.9
5%	3%	24%	46%	ratio 1 : 9.6 : 17.9
6%	3%	29%	55%	ratio 1 : 9.6 : 17.9
7%	4%	34%	64%	ratio 1 : 9.6 : 17.9
8%	4%	39%	73%	ratio 1 : 9.6 : 17.9
9%	5%	44%	82%	this study, 1 : 9.6 : 17.9
10%	5%	49%	91%	ratio 1 : 9.6 : 17.9
11%	6%	54%	100%	ratio 1 : 9.6 : 17.9

Supplementary Note S3. Technical details on mass spectrometer analyses at the Stable Isotope Facilities (SIF) at University of California, Davis (UC Davis)

The following text is extracted from the data reporting sheets and information available on the SIF website (https://stableisotopefacility.ucdavis.edu/ accessed 17-nov-2025).

1. $PO^{13}C$ and $PO^{15}N$ analyses using EA-IRMS

Sample preparation done by clients: Filters were dried at 50°C overnight, fumed over HCl, and thereafter encapsulated into tin capsules (6x6x12 mm, IVA176992027, Meerbusch, Germany).

Glass fiber filters were analyzed for ^{13}C and ^{15}N isotopes using an Elementar Vario Micro Cube elemental analyzer (Elementar Analysensysteme GmbH, Hanau, Germany) interfaced to a PDZ Europa 20-20 isotope ratio mass spectrometer (Sercon Ltd., Cheshire, UK). Samples were combusted at 1080°C in a reactor packed with chromium oxide and silvered copper oxide. Following combustion, oxides were removed in a reduction reactor (reduced copper at 650°C). The helium carrier then flows through a water trap (magnesium perchlorate and phosphorous pentoxide). CO_2 is retained on an adsorption trap until the N_2 peak is analyzed; the adsorption trap is then heated releasing the CO_2 to the IRMS.

During analysis, samples are interspersed with several replicates of at least four different laboratory reference materials. These reference materials have been previously calibrated against international reference materials, including: IAEA-600, USGS-40, USGS-41, USGS-42, USGS-43, USGS-61, USGS-64, and USGS-65) reference materials. The long-term standard deviation for natural abundance analysis is 0.2‰ for ^{13}C and 0.3‰ for N. The final delta and at% values are expressed relative to international standards VPDB (Vienna Pee Dee Belemnite) and Air for carbon and nitrogen, respectively.

2. DI^{13}C analyses using GasBench-IRMS

Sample preparation done by clients: Water samples were filtered through GF75 filter, filled headspace-free into glass vials (12 mL Exetainer®, #739W, Labco Limited, Wales, UK), preserved with zinc chloride ($ZnCl_2$, final conc. 0.05% w/v CAS: 7646-85-7, Merck) and stored at room temperature.

Dissolved inorganic carbon (DIC) in water is made suitable for stable isotope analysis by evolution to headspace CO_2 using phosphoric acid. Initially, 1 mL of 85 % H_3PO_4 is added to 12 mL Labco Exetainer vials, which are capped and evacuated to <10 mTorr and then flushed with helium at 500 mL min^{-1} for 20 seconds. Sample and reference materials are injected into the vials (volume added depends on DIC concentration), and the vials are held at room temperature overnight prior to isotopic measurement. Analysis of headspace CO_2 is performed in 12 mL Labco Exetainer vials on a Thermo Scientific GasBench II coupled to a Thermo Finnigan Delta Plus XL isotope-ratio mass spectrometer. One of every ten samples is analyzed in duplicate; further replicates may be analyzed if initial measurements fall outside expected measurement error.

Replicates of the quality control and assurance reference materials are measured every ten samples.

Calibration procedures for DIC are applied identically across reference and sample materials and are directly traceable to the primary isotopic reference material, Vienna PeeDee Belemnite (VPDB). First, a pure CO_2 reference gas is used to calculate provisional isotopic values of the sample peaks. Next, isotopic values are adjusted for changes in linearity and instrumental drift using a secondary reference material, Acros-2. Finally, measurements are scale-normalized to the primary reference material using secondary reference materials; Acros-2, NAU-1, NAU-2, and LSVEC. All secondary reference materials have been analyzed as carbonates and calibrated against three certified standard reference materials (i.e., NBS 18, NBS 19, and LSVEC) available from NIST and the IAEA. Final quality assessment is based on the accuracy and precision of the unbiased quality control materials, Scripps-B157 seawater, Fisher, SB-1, SB-2, and SB-3. Mean SD for natural abundance reference material replicates was ±0.30‰. Mean absolute accuracy for calibrated reference materials was within: ±0.16‰. Limit of quantification (LOQ), based on total peak area, is 3 V-s for $\delta^{13}C$.

3. ^{15}N-N_2 analyses using IRMS

Sample preparation done by clients: Water samples were gently filtered through GF75 filter to avoid bubble formation, filled immediately headspace-free into glass vials (12 mL Exetainer®, #739W, Labco Limited, Wales, UK), preserved with zinc chloride ($ZnCl_2$, final conc. 0.05% w/v CAS: 7646-85-7, Merck) and stored at room temperature.

Analysis of N_2 was performed using a Sercon CryoPrep gas concentration system interfaced to a Sercon 20-20 isotope-ratio mass spectrometer. Gas samples are purged from vials through a double-needle sampler into a helium carrier stream (20 mL/min). Then, N_2 is isolated and concentrated in preparation for isotopic analysis. First, N_2 gas is sampled by a rotary 8-port valve fitted with a 5–100 µL sampling loop and timed to capture the peak N_2 concentration in the carrier gas stream. This gas sample is passed to the IRMS through an Agilent molecular sieve 5A GC column (15 m x 0.53 mm ID x 50 µm film thickness, 40 °C, 2.2 mL/min).

Replicates of the quality control and assurance reference materials are measured every ten samples. Calibration and Reporting of Stable Isotope Ratios Calibration procedures for N_2 are applied identically across reference and sample materials and are directly traceable to the primary isotopic reference material (Air for $\delta^{15}N$). First, a

pure N_2 reference gas is used to calculate provisional isotopic values of the sample peaks. Next, isotopic values are adjusted for changes in linearity and instrumental drift. Finally, measurements are scale-normalized to the primary reference materials. The N_2 is calibrated against an Oztech N_2 standard. Final quality assessment is based on the accuracy and precision of $\delta^{15}N$-calibrated unbiased quality control materials UCDN2.

Mean measurement error and accuracy, as determined by replicate measurements of the quality control and assurance materials, must fall below expected measurement error (± 0.2 ‰ for $\delta^{15}N$). Accuracy and precision of the co- measured calibrated quality control and assurance materials are provided with data reports. Limit of quantification (LOQ), based on gas concentration, is 150 nmol per vial for N_2.

Mass spectrometry raw data are included in file Supplementary Data 1.

Supplementary Note S4. Calculating single-cell activities based on SIMS-derived data

Atom percent access (APE)

$$\mathrm{APE} = \left[\frac{\mathrm{R_f}}{\mathrm{R_f}+1} - \frac{\mathrm{R_i}}{\mathrm{R_i}+1}\right] \times 100\% \qquad \text{Eq. S1}$$

R_i – isotope ratio ($^{13}C/^{12}C$ or $^{15}N/^{14}N$) at the initial time point (T_0)

R_f – isotope ratio ($^{13}C/^{12}C$ or $^{15}N/^{14}N$) at the final time point (T_n)

The net incorporation (K_A) of an element as a function of the initial biomass was calculated following Stryhanyuk, et al. [14] and Pett-Ridge and Weber [15]

$$\mathrm{K_A} = \frac{\mathrm{R_f}-\mathrm{R_i}}{(1+\mathrm{R_i})\times(\mathrm{D_{gs}}\times(1+\mathrm{R_f})-\mathrm{R_f})} \qquad \text{Eq. S2}$$

D_{gs} - fraction of the rare isotope in the spiked pool [fx=R/(R+1)], equals fx_s

Cell-specific growth rates μ are calculated following Guillard [16] adapted to SIMS data.

$$\mu_{\mathrm{Cells}} = \frac{\ln(1+\mathrm{K_A})}{(\mathrm{t_n}-\mathrm{t_0})} \qquad \text{Eq. S3}$$

Element-specific incorporation rates (pmol C $cell^{-1}$ d^{-1} or pmol N $cell^{-1}$ d^{-1}) were calculated as (exemplary shown for nitrogen):

$$\text{Cell-specific N-fixation} = \mathrm{K_A} \times \text{cell-specific N-content} \qquad \text{Eq. S4}$$

References

1 Svedén, J. B., Adam, B., Walve, J., Nahar, N., Musat, N., Lavik, G., Whitehouse, M. J., Kuypers, M. M. M. & Ploug, H. High cell-specific rates of nitrogen and carbon fixation by the cyanobacterium *Aphanizomenon* sp. at low temperatures in the Baltic Sea. *FEMS Microbiol. Ecol.* **91**, (2015)

2 HELCOM. Guidelines for monitoring phytoplankton species composition, abundance, and biomass. (2021). https://helcom.fi/wp-content/uploads/2020/01/HELCOM-Guidelines-for-monitoring-of-phytoplankton-species-composition-abundance-and-biomass.pdf.

3 Olenina, I., Hajdu, S., Edler, L., Andersson, A., Wasmund, N., Busch, S. *et al.* Biovolumes and size-classes of phytoplankton in the Baltic Sea. *HELCOM Balt. Sea Environ. Proc.* **106**, 144, (2006)

4 Van den Wyngaert, S., Nawaz, A., Alacid, E., Wood-Rocca, S. M., Reñé, A., Garcés, E., Kremp, A. & Wurzbacher, C. Dynamics of zoosporic parasites in summer phytoplankton communities of the Baltic Sea. *FEMS Microbiol. Ecol.* **101**, (2025)

5 ggOceanMaps: Plot Data on Oceanographic Maps using ggplot2 (2024).

6 Wickham, H. *ggplot2: Elegant Graphics for Data Analysis*. (2016).

7 Pebesma, E. Simple Features for R: Standardized Support for Spatial Vector Data. *The R Journal* **10**, 439-446, (2018)

8 Pebesma, E. & Bivand, R. *Spatial Data Science: With Applications in R.*, <https://doi.org/10.1201/9780429459016> (2023).

9 Van den Wyngaert, S., Ganzert, L., Seto, K., Rojas-Jimenez, K., Agha, R., Berger, S. A. *et al.* Seasonality of parasitic and saprotrophic zoosporic fungi: linking sequence data to ecological traits. *The ISME Journal* **16**, 2242–2254, (2022)

10 Garg, R. & Maldener, I. The Formation of Spore-Like Akinetes: A Survival Strategy of Filamentous Cyanobacteria. *Microbial Physiology* **31**, 296-305, (2021)

11 Lee, S. & Fuhrman, J. A. Relationships between biovolume and biomass of naturally derived marine bacterioplankton. *Appl. Environ. Microbiol.* **53**, 1298-1303, (1987)

12 Klawonn, I., Bonaglia, S., Whitehouse, M. J., Littmann, S., Tienken, D., Kuypers, M. M. M., Brüchert, V. & Ploug, H. Untangling hidden nutrient dynamics: rapid ammonium cycling and single-cell ammonium assimilation in marine plankton communities. *The ISME Journal* **13**, 1960-1974, (2019)

13 Gerphagnon, M., Latour, D., Colombet, J. & Sime-Ngando, T. Fungal parasitism: Life cycle, dynamics and impact on cyanobacterial blooms. *PLOS ONE* **8**, e60894, (2013)

14 Stryhanyuk, H., Calabrese, F., Kümmel, S., Musat, F., Richnow, H. H. & Musat, N. Calculation of single cell assimilation rates from SIP-nanoSIMS-derived isotope ratios: A comprehensive approach. *Front. Microbiol.* **9**, 2342, (2018)

15 Pett-Ridge, J. & Weber, P. K. (2022). NanoSIP: NanoSIMS Applications for Microbial Biology. In: Ali Navid (ed). *Microbial Systems Biology: Methods and Protocols*. Springer US: New York, NY. pp 91-136.

16 Guillard, R. R. L. (1973). Division rates. In: Janet Stein (ed). *Handbook of phycological methods: Culture methods and growth measurement*. Cambridge University Press: Cambridge UK. pp 289-311.
