# Supplementary material for: Fungal parasites infecting N2-fixing cyanobacteria reshape carbon and N2 fixation and trophic transfer
Source: Nat Commun. 2026 Jan 2;17:154. doi: 10.1038/s41467-025-67818-x (PMC12775406; doi:10.1038/s41467-025-67818-x)
Supplement: Supplementary file 2 — Description of Additional Supplementary File [file 41467_2025_67818_MOESM2_ESM.pdf]

Description of Additional Supplementary File

File Name – Supplementary Data 1

Description: Raw data from mass-spectrometry analyses (SIMS: IMS1280 and NanoSIMS; EA-RIMS; IRMS).
