# Supplementary material for: Fungal parasites infecting N2-fixing cyanobacteria reshape carbon and N2 fixation and trophic transfer
Source: Nat Commun. 2026 Jan 2;17:154. doi: 10.1038/s41467-025-67818-x (PMC12775406; doi:10.1038/s41467-025-67818-x)
Supplement: Supplementary file 4 — Reporting Summary [file 41467_2025_67818_MOESM4_ESM.pdf]

nature portfolio

Corresponding author(s): Isabell Klawonn

Last updated by author(s): Dec 5, 2025

Reporting Summary

Nature Portfolio wishes to improve the reproducibility of the work that we publish. This form provides structure for consistency and transparency in reporting. For further information on Nature Portfolio policies, see our Editorial Policies and the Editorial Policy Checklist.

Statistics

For all statistical analyses, confirm that the following items are present in the figure legend, table legend, main text, or Methods section.

n/a	Confirmed	
☐	☒	The exact sample size (*n*) for each experimental group/condition, given as a discrete number and unit of measurement
☐	☒	A statement on whether measurements were taken from distinct samples or whether the same sample was measured repeatedly
☐	☒	The statistical test(s) used AND whether they are one- or two-sided<br>*Only common tests should be described solely by name; describe more complex techniques in the Methods section.*
☒	☐	A description of all covariates tested
☐	☒	A description of any assumptions or corrections, such as tests of normality and adjustment for multiple comparisons
☐	☒	A full description of the statistical parameters including central tendency (e.g. means) or other basic estimates (e.g. regression coefficient) AND variation (e.g. standard deviation) or associated estimates of uncertainty (e.g. confidence intervals)
☐	☒	For null hypothesis testing, the test statistic (e.g. *F*, *t*, *r*) with confidence intervals, effect sizes, degrees of freedom and *P* value noted<br>*Give P values as exact values whenever suitable.*
☒	☐	For Bayesian analysis, information on the choice of priors and Markov chain Monte Carlo settings
☒	☐	For hierarchical and complex designs, identification of the appropriate level for tests and full reporting of outcomes
☒	☐	Estimates of effect sizes (e.g. Cohen's *d*, Pearson's *r*), indicating how they were calculated

Our web collection on statistics for biologists contains articles on many of the points above.

Software and code

Policy information about availability of computer code

Data collection	WinImage4.8
Data analysis	RStudio (2024.12.0), cutadapt (v2.8), dada2 (1.34.0), silva_SSUfungi_nr99_v138_2_toGenus_trainset, pr2_version_5.1.0_SSU_dada2, decontam (1.26.0), DESeq2 (1.46.0), tidyverse, agricolae, pscl, MASS, Origin2022, Excel2019, ImageJ2 (v1.54f), NisElements

For manuscripts utilizing custom algorithms or software that are central to the research but not yet described in published literature, software must be made available to editors and reviewers. We strongly encourage code deposition in a community repository (e.g. GitHub). See the Nature Portfolio guidelines for submitting code & software for further information.

Data

Policy information about availability of data

All manuscripts must include a data availability statement. This statement should provide the following information, where applicable:

- Accession codes, unique identifiers, or web links for publicly available datasets
- A description of any restrictions on data availability
- For clinical datasets or third party data, please ensure that the statement adheres to our policy

Source data are provided with this paper. Sequence data have been deposited in ENA (European Nucleotide Archive) under project accession no. PRJEB96922 [https://www.ncbi.nlm.nih.gov/search/all/?term=PRJEB96922]. Accession numbers used to construct the phylogenetic tree in Supplementary Fig. 5 are listed in Supplementary Table S5, as reported in the NCBI database.

Research involving human participants, their data, or biological material

Policy information about studies with human participants or human data. See also policy information about sex, gender (identity/presentation), and sexual orientation and race, ethnicity and racism.

Reporting on sex and gender	na
Reporting on race, ethnicity, or other socially relevant groupings	na
Population characteristics	na
Recruitment	na
Ethics oversight	na

Note that full information on the approval of the study protocol must also be provided in the manuscript.

Field-specific reporting

Please select the one below that is the best fit for your research. If you are not sure, read the appropriate sections before making your selection.

☐ Life sciences ☐ Behavioural & social sciences ☒ Ecological, evolutionary & environmental sciences

For a reference copy of the document with all sections, see nature.com/documents/nr-reporting-summary-flat.pdf

Ecological, evolutionary & environmental sciences study design

All studies must disclose on these points even when the disclosure is negative.

Study description	Quantitative data. Natural sample.
Research sample	Baltic Sea water. Microbial plankton. Sampling on July 4th, 2022, 21:00, UTC+2 at the coast of the Southern Baltic Sea (Belt Sea, Heiligendamm, 54°08'46.7"N, 11°50'36.1"E.
Sampling strategy	Incubation in triplicates + one control. Statistical differences between two samples were calculated using the Mann–Whitney test for non-normally distributed data, the Welsh test for normally distributed data with non-equal variance, and the t-test for normally distributed data with equal variance. Normal distribution was verified using the Shapiro test and data variance with the F-test. Statistical differences between multiple groups were determined with Tukey's HSD test (for normally distributed data populations) and the Kruskal–Wallis test (if normal distribution was rejected, with Bonferroni correction for p-value adjustment). Count data of associated bacteria were dominated by zeros and thus modeled using a zero-inflated Poisson (ZIP) and Zero-Inflated Negative Binomial (ZINB) model. The model with the lower Akaike Information Criterion (AIC) was given preference. Tests were run in RStudio2024.12.0 using the packages tidyverse 73, agricolae 74, pscl 75,76, and MASS 77.
Data collection	Single-cell isotope probing, microscopy, and biogeochemical analyses by A.F., C.D.L, J.S., M.J.W., A.V., I.K.
Timing and spatial scale	One-time sampling on July 4th, 2022, 21:00, UTC+2 at the coast of the Southern Baltic Sea (Belt Sea, Heiligendamm, 54°08'46.7"N, 11°50'36.1"E.
Data exclusions	No data were excluded.
Reproducibility	Isotope-tracer incubations were done with four replicate bottles. Additional samples for determining infection prevalences were taken weekly at the sampling station Heiligendamm.<br>Reproducibility was ensured through detailed protocols, standardized laboratory procedures, and comprehensive lab documentation. However, environmental samples are inherently unique, as they represent a snapshot of dynamic natural conditions. Thus, while experimental workflows can be reproduced, exact environmental states cannot be recreated.
Randomization	Yes, samples were randomized during analyses to avoid batch effects.
Blinding	Isotope and biogeochemical analyses were blinded.

Did the study involve field work? ☒ Yes ☐ No

Field work, collection and transport

Field conditions	On July 4, 2022, field conditions were mild and typical for early summer—cool but stable, with air temperatures in the high teens, and aligned closely with long-term averages rather than any heatwave spike. Recorded temperature on that day between 15-22°C .
Location	Southern Baltic Sea (Belt Sea, Heiligendamm, 54°08'46.7"N, 11°50'36.1"E. ca. 1m water depth
Access & import/export	Sampling from pier.
Disturbance	No disturbance to report.

Reporting for specific materials, systems and methods

We require information from authors about some types of materials, experimental systems and methods used in many studies. Here, indicate whether each material, system or method listed is relevant to your study. If you are not sure if a list item applies to your research, read the appropriate section before selecting a response.

Materials & experimental systems

n/a	Involved in the study	
☒	☐	Antibodies
☒	☐	Eukaryotic cell lines
☒	☐	Palaeontology and archaeology
☐	☒	Animals and other organisms
☒	☐	Clinical data
☒	☐	Dual use research of concern
☒	☐	Plants

Methods

n/a	Involved in the study	
☒	☐	ChIP-seq
☒	☐	Flow cytometry
☒	☐	MRI-based neuroimaging

Animals and other research organisms

Policy information about studies involving animals; ARRIVE guidelines recommended for reporting animal research, and Sex and Gender in Research

Laboratory animals	Study did not involve laboratory animals.
Wild animals	Study did not involve wild animals.
Reporting on sex	Sex of the studied microorganisms was not considered.
Field-collected samples	We conducted laboratory work (isotope-tracer incuabtions) with field-sampled planktonic microbes. Sample date and location: July 4th, 2022, 21:00, UTC+2) at the coast of the Southern Baltic Sea (Belt Sea, Heiligendamm, 54°08'46.7"N, 11°50'36.1"E Incubations lasted for 0 hours (start), 10 hours in darkness (10 h), and 10 hours in darkness followed by 11 hours in light (21 h) inside a growth chamber (KBW 400, BINDER, Germany) at 18°C with 0 µE m-2 during darkness and 40 µE m-2 during light.
Ethics oversight	No ethical approval or guidance was required for work with environmental planktonic microorganisms because they are not vertebrates, not animals covered by animal-welfare legislation, not human/clinical subjects, nonhazardous, and not genetically modified organisms. Sample were taken from national waters (Nagoya protocol or similar not required).

Note that full information on the approval of the study protocol must also be provided in the manuscript.

Plants

Seed stocks	na
Novel plant genotypes	na
Authentication	na
